# Supplemental Intervention for Alcohol Use Disorder Treatment Patients With a Co-Occurring Anxiety Disorder: Technical Development and Functional Testing of an Autonomous Digital Program

**DOI:** 10.2196/62995

**Published:** 2024-12-31

**Authors:** Linda Marie Rinehart, Justin Anker, Amanda Unruh, Nikki Degeneffe, Paul Thuras, Amie Norden, Lilly Hartnett, Matt Kushner

**Affiliations:** 1 Department of Psychiatry & Behavioral Sciences, University of Minnesota, Minneapolis, MN, United States; 2 Minnesota Veterans Affairs Medical Center, Minneapolis, MN, United States; 3 Carlson School of Management, University of Minnesota, Minneapolis, MN, United States

**Keywords:** alcohol use disorder, anxiety disorder, comorbidity, digital intervention, psychological treatments, addiction, community-based practice, therapy, stress, depression, therapist-delivered therapies

## Abstract

**Background:**

Anxiety disorders are common in alcohol use disorder (AUD) treatment patients. Such co-occurring conditions (“comorbidity”) have negative prognostic implications for AUD treatment outcomes, yet they commonly go unaddressed in standard AUD care. Over a decade ago, we developed and validated a cognitive behavioral therapy intervention to supplement standard AUD care that, when delivered by trained therapists, improves outcomes in comorbid patients. However, this validated intervention, like many others in addiction care, has not been taken up in community-based AUD treatment programs. This phenomenon—empirically validated treatments that fail to be widely adopted in community care—has been termed the “research-to-practice gap.” Researchers have suggested that the availability of fully autonomous digital equivalents of validated therapist-delivered therapies could reduce some barriers underlying the research-to-practice gap, especially by eliminating the need for costly and intensive therapist training and supervision.

**Objective:**

With this in mind, we obtained a Program Development Grant (R34) to conduct formative work in the development of a fully autonomous digital version of our previously validated therapist-delivered intervention for AUD treatment patients with a comorbid anxiety disorder.

**Methods:**

In the first phase of the project, we developed the digital intervention. This process included: (1) identifying appropriate collaborators and vendors; (2) consultation with an e-learning expert to develop a storyboard and accompanying graphics and narrative; (3) video production and editing; and (4) interactive programming. The second phase of the project was functional testing of the newly developed digital intervention conducted in 52 residential AUD treatment patients with a comorbid anxiety disorder. Patients underwent the 3 one-hour segments of the newly developed intervention and completed user surveys, knowledge quizzes, and behavioral competence tests.

**Results:**

While the development of the digital intervention was successful, the timeline was approximately double that projected (1 vs 2 years) due to false starts and inefficiencies that we describe, including lessons learned. Functional testing of the newly developed digital intervention showed that, on average, patients rated the user experience in the upper (favorable) 20% of the response scales. Knowledge quizzes and behavioral demonstrations showed that over 80% of participants gained functional mastery of the key skills and information taught in the program.

**Conclusions:**

Functional testing results in this study justify a randomized controlled trial of the digital intervention’s efficacy, which is currently ongoing. In sharing the details of our challenges and solutions in developing the digital intervention, we hope to inform others developing digital tools. The extent to which the availability of empirically validated, fully autonomous digital interventions achieves their potential to reduce the research-to-practice gap remains an open but important empirical question. The present work stands as a necessary first step toward that end.

## Introduction

Epidemiological studies consistently find that individuals with alcohol use disorder (AUD) experience an anxiety disorder at approximately double the rate found in the general population (“comorbidity”) [1]. Lending clinical importance to this observation is the elevated risk for relapse following AUD treatment found in the large subgroup of comorbid AUD treatment patients [2,3]. Kushner et al [4] developed and validated a cognitive behavioral therapy (CBT)–based intervention designed to supplement standard AUD care for individuals with a comorbid anxiety disorder to improve AUD outcomes for this large subpopulation [4,5].

The CBT intervention focuses on 3 primary elements: psychoeducation, cognitive restructuring, and coping skills (including breathing-based relaxation and training in systematic problem-solving). The psychoeducation and skills are introduced within the context of the “vicious cycle” model of comorbidity, ie, the interacting cognitive, behavioral, and physiological processes that mutually exacerbate both anxiety and drinking as part of an interactive feedback system. The presented material is interspersed with brief exercises to help the patient learn how to apply the presented material and to allow patients to ask questions at pre-established points.

Early in the therapy, each patient identifies a recent and ongoing problem they can use to practice or apply the skills and concepts being taught that ideally include the following elements: (1) is recent (ideally having occurred in the past 2 weeks); (2) is common for them (ideally a problem that has occurred at least 3 times in the past 3 months); (3) is likely to happen again in the near future (ie, is an ongoing problem); and (4) has commonly involved strong negative affect and a strong urge to drink, whether or not drinking occurred. Each skill component is explained conceptually within the context of the vicious cycle of comorbidity, then demonstrated using a stock example and, finally, practiced by the patient using the problem situation they identified to work on. Most of these exercises include a worksheet the patient is asked to fill in. Standardized written homework assignments with an accompanying worksheet are given at the end of each session. The approximately 1-hour sessions are conducted (as far as possible) on consecutive business days in the late afternoon, which is a period of free time in the residential AUD treatment.

Kushner et al [4] found that standard AUD treatment supplemented by this brief therapist-delivered intervention resulted in superior alcohol outcomes compared to standard AUD treatment alone [4]. Unfortunately, the uptake of empirically validated therapies (including the therapy described above) in community-based treatment programs remains limited [6-10]. This phenomenon is commonly referred to as the “research-to-practice gap.” The research-to-practice gap has raised concerns that the research field’s success in developing and validating novel therapies is out of balance with its limited capacity to embed these treatments into community practice [11-13].

Digital programs that can autonomously deliver empirically supported interventions could reduce the research-to-practice gap by eliminating the need for lengthy and expensive staff training and expert supervision while minimizing disruptions to the delivery of standard care [14]. Based on this premise, we obtained a National Institutes of Health (NIH) Planning Grant Program (R34) to conduct formative work to develop and establish the functionality of an autonomous digital version of our therapist-delivered CBT for comorbid AUD treatment patients.

This report aims to (1) describe the process of developing the digital intervention, including challenges, solutions, and lessons learned and (2) present data related to patient user experience, knowledge acquisition, and skills mastery.

## Method

### Development of the Digital Therapy

#### Collaborators and Vendors

**Figure 1.**
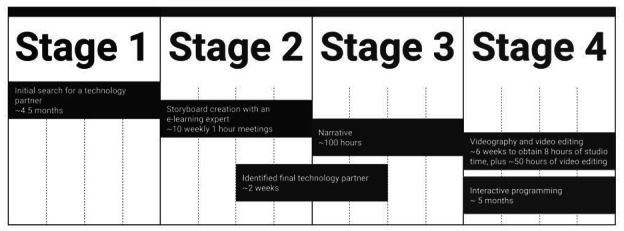
Timeline for developing digital intervention.

Our core lab team includes clinical addiction researchers, statisticians, addiction therapists, lab manager or coordinators, and research assistants. However, we found it necessary to identify and recruit several specialists to develop the program. As described in detail below, our early encounters with technologists were less productive than they might have been due to our lack of a coherent model for communicating the program’s contents. This problem was addressed by the addition of an e-learning specialist (author, AN), who aided us in conceptualizing the digital intervention as an educational endeavor (ie, teaching the concepts and application of CBT skills). Conceptualizing the program as a learning tool informed other important decisions, including the use of an LMS service provider to host the program. Here it was determined that teaching CBT skills via an autonomous internet system requires all the interactivity and flexibility of a remote electronic college course. This interactivity requires an LMS infrastructure with technical support. We also adopted a hybrid approach to managing some technical elements such as programming. Here we developed in-house proficiency in video editing while contracting expertise to accomplish the programming (eg, JavaScript coding) and unique animation needed for our purposes. Finally, recording of the narrator for integration with the graphics required a professional greenscreen studio and professional videography services provided by a University of Minnesota-based vendor. Figure 1 shows the steps and approximate timeline for acquiring these collaborators and conducting the relevant work, which is discussed in detail in the following sections.

#### Initial Search for a Technology Partner

We initially thought that the therapist-delivered CBT program provided sufficient structure to allow for the outsourcing of the digital conversion with little additional planning or decision-making on our part. Our unsuccessful interactions with several potential technology partners, however, ultimately led us to conclude that we currently lacked the expertise to formulate a sufficiently refined vision and organizational structure to engage effectively with a technology company in terms of how we wanted the program formatted. This lack of vision, we believed, led companies to either impose their own structure based on their existing digital tools or to look to us to provide a detailed structure that we were not sufficiently prepared to offer. This analysis and the conclusions led us to seek additional consultation to help us define how our interactive therapist-delivered therapy could be optimally structured for an autonomous digital format.

#### Storyboard Creation With an e-Learning Expert

After considering several possibilities, we ultimately analogized our task of translating a therapist-based therapy to a digital intervention to that of converting an instructor-led class to an autonomous digital class. Based on this, we sought input from a senior consultant for instructional design and educational technologies at the University of Minnesota Carlson School of Management (author AN), who guided us through transforming in-person lecture-based classes into remote autonomous teaching programs.

These consultations were done over approximately 10 weekly meetings with the primary goal of “storyboarding*”* the therapy content. The storyboarding process began by breaking the content within each of the 3 sessions into naturally cohering content “chapters*,*” each to be initially developed separately. Each chapter underwent a “brainstorming” phase in which the key concepts and skills were mapped logically. Initially, this sequence was represented by hand drawings with no (or minimal) text to evaluate the flow and inform the graphic elements needed for the chapter. Next, metrics were developed for all specific knowledge and skill concepts within chapters to assess their successful acquisition, ie, specific activities the user could engage with to demonstrate that they correctly understood and could apply the concepts and skills. Although we had initially thought of using the PowerPoint graphics from the therapist-delivered therapy verbatim in the digital version, these original graphics now served only as a starting point for the storyboarded chapters developed for the digital program. For example, while the therapist-delivered slides were text-heavy, the e-learning expert emphasized the importance of developing simple graphics conforming to our newly developed storyboard that were engaging, captured the point being made in a simple and occasionally humorous way, and that greatly minimized or eliminated text. We used open-access images or original graphics generated using PowerPoint where possible. However, several graphics we envisioned required hand drawing, for which we used a graphic artist.

#### Narrative

Just as we had mistakenly thought we could directly transfer the slides from the therapist-delivered therapy to the digital program, we initially thought that the narrative for the digital therapy could be directly taken from the talking points embedded in the therapist-delivered slides. However, this plan proved untenable because of changes to the graphics and structural rollout of the material resulting from our restructured storyboard, as well as the need for increased efficiency and clarity in a digital program that, unlike the therapist-delivered version, could not be tailored to the needs of specific patients (eg, those with less ability to grasp the material). With some preliminary attempts at presenting the narrative for the digital program extemporaneously (with minimal bullet point notes), we deemed that this approach did not result in a smooth and tightly focused product (eg, excessive pauses, digressions, repetitions). Under the further guidance of our e-learning collaborator, we created verbatim scripted narratives for the entire program to be read from a teleprompter after sufficient practice that allowed the language to sound extemporaneous using common conjunctions and natural patterns of speech. As the developer of the intervention, the lead investigator (MGK) produced the narration script. Creating the verbatim scripted narrative for the entire 3 hours of programming (around 30,000 words) required approximately 100 hours to complete.

#### Videography and Video Editing

We designed the digital program so that the narrator would be visible in the inset box on the screen, and the appropriate graphic would be displayed prominently. This required an audio-video recording of the narrator against a green screen delivering the narrative so that it could be edited into the graphical content of the program. Initially, we purchased commercially available recording equipment and a greenscreen to produce these recordings in-house. However, this approach produced suboptimal audio and visual results. This led us to consult with a professional videography service housed at the University of Minnesota. Here, we learned that professional quality equipment (audio, visual, and lighting) and trained professionals were needed to produce a professional quality product. Based on this, we contracted with the university-based audio or visual service to produce the greenscreen recordings. Using their professional-grade equipment, studio, and 2 staff (1 sound and 1 video), we engaged with this service to record 2 versions of the narrator reading the narrative from a teleprompter, allowing them to edit a final optimal version. Because of the challenges of obtaining approximately 8 hours of studio time and the multiple staff needed to produce these recordings, we made the recordings over multiple studio sessions whenever studio and staff time were available. This process took 6 weeks to complete, not including the time we had already spent working on the problem in-house without success. Following the successful recording, the raw footage was imported, the greenscreen background was removed, and the video of the narrator was integrated precisely with the graphics. This effort, requiring approximately 50 hours, was done in-house using Adobe Premiere Pro for video editing (author, AU).

#### Interactive Programming

Once the content organization, graphics, narrative, and exercises were fully developed, it was no longer difficult to identify an appropriate technology partner to host the program material and program it for display on an internet-based platform with secure data capture. Because the program requires all the interactivity and flexibility of a remote electronic class, we chose to host it on the Internet via a learning management system (LMS) platform. The LMS uses Sharable Content Reference Model files to accomplish interactive educational technology, allowing for communication between a user and the host system and to package transferable zip files (data capture) in a *Package Interchange Format*.

In consultation with this technology partner, one of our team members (ND) developed proficiency with Adobe Captivate to program the interactive components of the program. This included data capture and carryforward for inputs related to particular skills and interactive quizzes with frequent feedback throughout the program. The technology partner provided the JavaScript coding needed to allow these materials to be accessed and executed seamlessly on the LMS platform securely and reliably.

#### Description of the Digital CBT Program

The digital program conveys the same primary skill elements as the therapist-delivered version as guided by an “expert narrator” (the last author), who is visible on screen as graphics accompany the narration. Approximately every 5 minutes, the presentation of new material is interrupted with a brief “knowledge quiz.” These quizzes were designed to correspond to the therapist version in which patients are asked to describe the content presented in their own words with corrections by the therapist as needed and to diminish monotony from an uninterrupted narrative. In the digital version, these quizzes typically involved 2-4 true or false questions designed to be fairly easy if the patient was paying attention and generally understood the material presented. Feedback is provided immediately on the screen. If the answer is incorrect, the system describes the correct answer and why. In addition to the quizzes, each primary skill element is practiced within the session. These practice exercises follow the same format: (1) the narrator explains the skill and how it is designed to disrupt a particular element in the vicious cycle; (2) the narrator demonstrates the proper use of the skill element using a stock example; (3) the patient is given a brief digital quiz to evaluate their understanding of the skill’s purpose and execution; and (4) the patient is guided to practice the skill as applied to the personalized problem that they had identified at the beginning of the program. Interactive forms on the screen allow the patient to move through worksheets as they would have using hard copies in the therapist-delivered version. The forms also allow for data capture and carry forward information gathered earlier in the program (eg, the patient’s individualized problem is retained and displayed for each new skill practiced). Homework is given at the end of each session, and hardcopy forms are printed out (the patients do not have access to internet-enabled equipment in their residential program outside of the study session). A study staff member collects it at the beginning of the following session. Patients rate their degree of understanding and perceived usefulness of the material presented after each session.

### Functionality Testing of the Digital CBT Therapy

#### Recruitment

Patients were recruited from AUD treatment patients in the M-Health Fairview’s “Lodging Plus” (LP) 28-day adult residential addiction treatment program in Minneapolis, Minnesota. Recruitment procedures entailed posting an announcement about study participation in the treatment unit and inviting interested patients to complete a brief screening form. Study staff reviewed the completed screening forms to determine who would likely meet the study’s inclusion criteria. These patients were invited for further screening, and if qualified, a baseline assessment was obtained.

#### Inclusion and Exclusion Criteria

Inclusion criteria were a current diagnosis of AUD, at least one current (ie, past month) diagnosis of either panic disorder, social anxiety disorder, or generalized anxiety disorder (all using Diagnostic and Statistical Manual of Mental Disorders, Fourth Edition criteria), and aged between 18 and 65 years. Exclusion criteria were the presence of psychosis, including mania, acute suicidality, inability to read or speak English, court-ordered treatment, or cognitive impairment deemed detrimental to providing informed consent or the inability to fully participate in the study. Patients were not excluded if they met diagnostic criteria for major depression or post-traumatic stress disorder.

#### Assessment Appointments

The baseline and post-treatment assessments occurred while participants were in the LP residential AUD program. The baseline assessment typically occurred between the first and second week in the LP program, and the post-treatment assessment occurred on the first business day following completion of the digital intervention (described below) and always prior to discharge from the LP program.

#### Interviews, Assessments, and Self-Report Measures

Recent alcohol use (past 120 d) was assessed at baseline using the Timeline Follow-Back interview. Diagnoses for inclusion and exclusion were obtained using the Structured Clinical Interview for DSM-IV [15]. Anxiety symptoms were quantified using the Spielberger Trait Anxiety Inventory [16]. Depression symptoms were quantified using the Beck Depression Inventory (BDI) [17]. Demographic information was obtained via the investigator-developed Demographics Questionnaire*.* Program-Related Knowledge Acquisition was assessed with investigator-developed quizzes presented approximately every 5 minutes during the presentation of new material in the computer-delivered program. Quizzes typically involved 2-4 true or false questions designed to be “easy” if the patient was paying attention and generally understood the material presented. User satisfaction ratings were obtained using investigator-developed surveys after each session (session-specific) and at the end of the program (program-specific). Participants responded to questions such as ease of use, satisfaction with the program, and the likelihood of recommending the program to others. Response options for session-specific questions were obtained on a number scale with lower numbered ratings, which meant a less favorable response.

#### Behavioral Skills Acquisition Assessments

Participants completed post-therapy skills demonstration tests to assess program-related skills acquisition. To standardize the test, participants were presented with the same hypothetical scenario (ie, they are having financial problems and are overdue on rent, and, as a result, they are experiencing significant anxiety accompanied by a strong urge to drink). Based on this scenario, participants were asked to apply cognitive restructuring and problem-solving skills using the same worksheets used to teach these skills in the program. Participants were also evaluated during a 30 seconds paced-diaphragmatic breathing exercise, which had been taught in the program. In total, 2 licensed psychologists independently scored each participant’s completed cognitive-restructuring (6 elements) and problem-solving (4 elements) worksheets, and a trained research assistant scored participants’ performance on the breathing exercise (2 elements). Each element was scored as either: (1) “as taught,”; (2) “clinically satisfactory but not fully as taught,”; or (3) “clinically unsatisfactory” (this rating was also given for non-responses). After the 2 independent raters scored the cognitive restructuring and problem-solving worksheets, their ratings were compared. For the ratings of cognitive restructuring worksheets, there were a total of 252 ratings with 36 disagreements for a mean κ of 0.78 (SD 0.08). For the problem-solving worksheets, there were 152 ratings with 17 disagreements for a mean κ of 0.80 (SD 0.12). All rating disagreements were then resolved by consensus of the 2 raters. Using these final ratings, participants were deemed to have gained a functional use of the skill if their average rating across the skill elements was 2 or greater (ie, at least clinically satisfactory). The diaphragmatic breathing demonstration was scored based on a single rater’s observation of (1) breathing from the area of the diaphragm (ie, movement in the stomach with little or no movement of chest and shoulders for both inhalation and exhalation) and (2) pace (holding each inhalation and exhalation for approximately 2 seconds).

### Ethical Considerations

This study was approved by the University of Minnesota’s Institutional Review Board on May 22, 2017 (STUDY00000262), prior to the initiation of recruitment, and each participant provided informed consent to participate. Study data are de-identified, and all data is stored securely using encryption according to university policy for protection of confidentiality. Participants were compensated using debit cards loaded with money following the completion of each study element. Participants were compensated US $25 for the baseline and post-treatment assessments, US $10 for each day of study intervention, and an additional US $5 if homework was completed and returned.

## Results

### Participant Characteristics

We recruited 52 individuals meeting the study’s criteria. Table 1 shows the demographic and clinical characteristics of the sample. Of the 52 who consented to the study, 45 (87%) completed the computer-delivered intervention. In total, 3 participants dropped out before starting the intervention (ie, one was discharged early from LP before starting the intervention sessions, and 2 did not respond to requests to schedule appointments for the intervention sessions after being consented). In total, 4 participants dropped out after completing at least one intervention session (ie, 3 participants were discharged from the LP program before completing all 3 intervention sessions, and 1 participant who had started the intervention did not respond to requests to schedule additional appointments).

**Table 1.** Baseline demographic and clinical characteristics of the sample (N=52).

	Baseline sample
Male, n (%)	28 (54)
Age in years, mean (SD)	42.87 (10.79)
Race or ethnicity, n (%)	
White, non-Hispanic	38 (73)
Black	6 (12)
Hispanic	2 (4)
Native American	0 (0)
Asian	2 (4)
Other	4 (8)
Any illicit drug use, n (%)	32 (62)
Percent drinking days, mean (SD)	60.03 (34.90)
STAI^a^, mean (SD)	57.08 (9.19)
BDI^b^, mean (SD)	27.98 (8.63)
GAD^c^, n (%)	46 (88)
PD^d^, n (%)	24 (47)
SAD^e^, n (%)	37 (72)
>1 anxiety diagnosis, n (%)	40 (77)

a STAI: Spielberger Trait Anxiety Inventory.

b BDI: Beck Depression Inventory.

c GAD: generalized anxiety disorder.

d PD: panic disorder.

e SAD: social anxiety disorder.

### Satisfaction Ratings

Participant satisfaction ratings administered at post-treatment and for each session are shown in Table 2 and Table 3. Ratings following each session were generally high. At post-treatment, on a scale with a maximum rating of 10, the mean “satisfaction” score for the program overall was 8.87 (SD 1.12), and the mean response to “the program was helpful to you” was 8.67 (SD 1.44). When asked if they would recommend the program to others, the mean rating was 8.93 (SD 1.27).

**Table 2.** Satisfaction with each session of the computer-delivered cognitive behavioral therapy intervention.

	Session 1 (n=49), mean (SD)	Session 2 (n=47), mean (SD)	Session 3 (n=45), mean (SD)
I understood material presented (1-10)	9.73 (0.57)	8.38 (1.66)	8.87 (1.34)
The program was engaging (1-10)	9.16 (1.05)	8.53 (1.65)	8.64 (1.30)
The skills and examples applied to my symptoms (1=not at all; 5=extremely well)	4.43 (0.61)	4.15 (0.81)	4.00 (0.56)
To what degree was the information and skills new and different from what you’ve learned in Lodging Plus? (1=not at all new; 5=completely new)	3.71 (1.04)	4.23 (0.87)	4.20 (0.97)

**Table 3.** Satisfaction with the computer-delivered cognitive behavioral therapy intervention overall at posttreatment assessment (n=45) (0=not at all; 10=very much)

Question	Response, mean (SD)	Mode	Range
How logical (makes good sense) do you think the program is?	9.07 (1.00)	10	6‐10
I would plan to use the tools and information I learned in the program frequently.	8.58 (1.34)	10	6‐10
I thought the program was easy to use.	8.32 (1.67)	10	3‐10
How satisfied are you with the program?	8.87 (1.12)	10	6‐10
To what extent do you feel confident recommending this program to a friend who had the same problems?	8.93 (1.27)	10	5‐10
To what extent do you think the program was helpful to you?	8.67 (1.45)	10	4‐10

### Program Knowledge and Skills Competency

Participant scores on knowledge quizzes given during each session and homework completion following each session are presented in Table 4. Participants’ within-session scores on knowledge quizzes indicated a good understanding of the material presented (>80% correct answers) at all sessions. Similarly, homework completion was high across all sessions (>80%).

**Table 4.** Participant scores on knowledge quizzes administered during each computer-delivered cognitive behavioral therapy session and homework completion following each session.

	Session 1	Session 2	Session 3
Within-session comprehension on quizzes (% correct), mean (SD)^a^	95.8 (6.3)	88.8 (5.5)	84.1 (8.6)
Homework completion, n (%)^b^			
Fully complete	46 (98)	41 (89)	29 (81)
Partially complete	0	2 (4)	0 (0)
Incomplete	1 (2)	3 (7)	7 (19)

a Session 1: n=49; session 2; n=47; session 3: n=44.

b Session 1: n=47; session 2: n=46; session 3: n=36.

Mastery of cognitive restructuring, systematic problem-solving and diaphragmatic breathing was assessed using the scores from trained raters. Functional mastery of the 3 primary skills was determined as having been met when the mean rating of the skill’s elements was 2 or greater (recall that scores of 1=unsatisfactory, 2=satisfactory for clinical purposes but not fully as taught, 3=as taught). Specifically, a total of 18 points was possible for cognitive restructuring (ie, 6 elements each with a maximum score of 3) with mastery established by a score of 12 or greater (ie, an average score of at least 2 “satisfactory” across 6 skill elements). In total, 12 points were possible for the problem-solving exercise (4 elements, each with a maximum score of 3), with functional mastery established with a score of 8 or greater. A total of 6 points were possible for the breathing exercise (2 elements, each with a maximum score of 3), with functional mastery established by a score of 4 or greater. Table 5 shows that over 90% achieved functional mastery in cognitive restructuring and breathing, and over 80% achieved functional mastery in problem-solving.

**Table 5.** Clinical benefit of computer-delivered cognitive behavioral therapy applied in “real world” situations (objective criteria).

	Cognitive restructuring (n=42)	Systematic problem-solving (n=38)	Diaphragmatic breathing (n=44)
Scores, mean (SD)	14.90 (3.04)^a^	9.63 (2.38)^b^	5.39 (0.94)^c^
Does not meet criteria, n (%)	4 (10)	7 (18)	2 (5)
Meets criteria, n (%)	38 (91)	31 (82)	42 (95)

a Maximum score=18

b Maximum score=12

c Maximum score=6

## Discussion

### Principal Findings

To make our therapist-delivered intervention for comorbid AUD patients more easily adopted and scaled for use in community settings, we sought and obtained an NIH Planning Grant Program (R34) to conduct the formative work described in this paper. Here, we provide details surrounding the development of the digital intervention to inform interested researchers conducting similar work. Testing of the newly developed digital intervention supports its functionality in the patient population for which it was intended, ie, individuals undergoing standard residential AUD treatment while experiencing a co-occurring (“comorbid”) anxiety disorder. On average, participants rated the program as logical, easy to use, and relevant to their problems. Moreover, most indicated that the program provided information and skills not provided in their standard AUD treatment. Additionally, knowledge and skills evaluation showed that most participants learned the key information presented in the program and gained functional mastery of the skills taught. Although this study was not designed to establish the efficacy of the digital therapy, these foundational findings were sufficiently positive to enable us to obtain new NIH funding to conduct a randomized clinical trial of the digital intervention, which is currently ongoing.

A small but growing number of high-quality studies have demonstrated the feasibility of translating highly structured therapist-delivered programs (eg, CBT) to computer-delivered platforms for a variety of specific conditions [18-26]. Still, few studies of digital therapeutics aimed at addiction have addressed common comorbid psychiatric conditions [27-29], such as anxiety disorder [30]. This is an important omission since rank-in-file addiction counselors who are less well trained to address common comorbidities in addiction might view a fully autonomous digital program such as ours as more valuable than digital programs that deliver therapies counselors are already using in their standard practice. In fact, such redundant programs might even pose a threat to counselors if they thought their standard interventions could be outsourced to a digital program.

In considering digitally delivered interventions for addiction, Budney et al [31] outlined numerous potential benefits such interventions could offer. One such benefit is the capacity to deliver interventions with perfect fidelity; ie, each administration conforms perfectly to the developers’ intentions and conforms precisely to its administration in studies upon which the intervention’s validity is based. This is not only a clinical benefit but also allows other researchers to introduce the intervention into their research without requiring expert training and supervision from the developers. Another advantage of digital interventions is the cost savings associated with hiring, training, and supervising live therapists to conduct new interventions. Obviating the need for specially trained and supervised interventionists could reasonably be expected to reduce the barriers to adopting new interventions. However, because our intervention was developed as an “add-on” for comorbid patients undergoing standard AUD treatment, the potential to benefit individuals for whom AUD treatment is not accessible is still unknown.

### Limitations

We were inspired to conduct this foundational research by the large research-to-practice gap in the addiction field, including our own empirically validated intervention for comorbid AUD. While digital interventions are cheaper and easier to adopt relative to therapist-delivered equivalents (eg, obviates specialized therapist training and supervision), we are not aware of hard empirical studies evaluating the extent to which digital interventions are more likely to be adopted in community-based AUD care relative to live therapist-delivered interventions. Such applied questions, while central to justifying our work, cannot be addressed until a digital intervention is developed and shown to be functional. Therefore, the present work and the RCT testing of the digital intervention we recently initiated based on the work presented here are necessary prerequisites to learning how the availability of effective digital equivalents of therapist-delivered interventions affects its adoption. As noted above, the fact that comorbidity presents a unique challenge to classically trained addiction counselors could make the adoption of a digital version more attractive than digital versions of therapies that addiction counselors are already familiar with.

The characteristics of our intervention might limit the generalizability of our approach to digitization relative to other types of interventions. For example, CBT, the modality upon which our intervention was based, lends itself to the “learning-based” educational approach we used to a greater degree than many other therapeutic approaches that rely less on communicating knowledge and skills and more on dynamic interactions with a therapist. There may also be important differences between developing a digital intervention based on an existing therapist-delivered therapy as we did versus developing a new digital intervention unrelated to an existing intervention. Further, most of our sample identified as White and non-Hispanic, limiting the ability to generalize our findings to diverse populations.

While consistent with the limited goals of this formative research, the absence of a control group and suitable outcome data in this study is a scientific limitation. For example, we could not determine how the user ratings we obtained for the digital intervention would compare to the ratings of a different intervention or the same intervention delivered by a live therapist. Similarly, we could not judge if the intervention had a clinically significant therapeutic effect relative to these comparison conditions. That said, we are conducting a more rigorous clinical trial that builds on this foundational work to answer these and related scientific questions.

Finally, while we have tested the therapist-delivered version of the CBT earlier [5], including it here as a comparison group was beyond the scope and aims of this formative work. We had considered blending the data from the earlier work to allow for a quasi-experimental empirical comparison between the 2 delivery methods but did not do so due to multiple threats to the validity of this approach, including: (1) the earlier study was conducted over a decade ago, leaving it vulnerable to unknown historical confounds; (2) a known historical confound is that the AUD residential program from which we recruit has increased the duration of treatment since the earlier study; and (3) the measures from the 2 studies, while highly similar, used different wording and response scales in many cases.

### Conclusions

The present study demonstrates the viability of translating a highly structured therapist-delivered CBT intervention for comorbid AUD treatment patients to a fully autonomous computer-delivered digital CBT intervention. However, the development process entailed a steep learning curve. This paper details our challenges, solutions, and lessons learned that could help other researchers seeking to translate an existing intervention into an autonomous digital equivalent. Participants reported being highly satisfied with the program, and most participants successfully gained the key knowledge and skills mastery the intervention was designed to impart. Once validated as a clinical intervention, the computer-delivered CBT has the potential to increase access to this highly specialized intervention and overcome barriers that contribute to the research-to-practice gap in addiction care.

## Abbreviations

AUD: alcohol use disorder
CBT: cognitive behavioral therapy
LMS: learning management system
NIH: National Institutes of Health
